# Surgery of the Vermiform Appendix

**Published:** 1895-05-11

**Authors:** 


### Progress in Surgery.

### SURGERY OF THE VERMIFORM APPENDIX.

*(Continued from page 172, Vol. XVII)*

**Appendicitis.**—Sonnenburg,[1] in writing of the pathology and treatment of parasyphilitis, arranges his seventy-seven cases under three heads. The first includes five cases of simple appendicitis operated upon with success. The second class comprises fifty-two cases of operations for perforating appendicitis without complications. All the patients recovered, and not one has hitherto had a relapse. In twenty-two cases the appendix was resected, but pus was invariably found, even when the operation was performed on the second day after the onset of the disease. The earlier the operation could be performed the easier was it to find and resect the appendix, the smaller was the focus, and the more rapidly recovery took place. The third class includes twenty operations for appendicitis with generalised peritonitis. These twenty cases are subdivided into two groups, the first of which is made up of eight cases of progressive fibrino-suppurative peritonitis; three of these died. Operation is, therefore, indicated in such cases, though there is always the risk of the surgeon being unable to open all the purulent foci. The remaining twelve operations forming the second group were for cases of diffuse septic peritonitis with symptoms of infection. All twelve died, and for this reason Professor Sonnenburg has given up operating in cases of this nature. This is an additional reason for operating early, so as to avoid this grave complication, in the presence of which surgical skill is powerless. Lennander[2] reports fifty-four operations, with three deaths. In fifteen cases laparotomy with extirpation of the appendix was performed, in twenty-one cases peri- or para-syphilitic abscesses were opened. In seventeen cases the appendix was removed during the period between relapses. In one case no vermiform appendix was found, although the cæcum could be entirely isolated. The author classifies as surgical cases those in which are found acute diffuse peritonitis and intra-peritoneal abscesses following perforation of the appendix; all parasyphilitic cases as soon as the presence of pus is diagnosticated; also those cases in which, through slow ulceration, a perforation is produced, though the diagnosis in these cases is difficult. Medical cases he considers to be only those of appendicular colic and all acute catarrhal processes within the appendix, and that remain within it, except, perhaps, those that produce a sero-fibrinous or simple fibrinous peritonitis. N. Jacobson[3] thinks that the only safe rule to follow is that evidence of continued progression at the end of twenty-four to thirty-six hours justifies and, indeed, calls for operation. Even the cases remaining stationary during this period must be viewed with suspicion. He quotes cases which indicate that there may be mitigation of all, and complete disappearance of most, of the symptoms, and yet, during the period of their subsidence, the diseased process has gone on steadily. The pulse may become lowered, the temperature sink to normal, and yet gangrene may be advancing or ulceration imminent. An increase of tenderness, the appearance of tumefaction, the development of a tumour, mean much more than any variation in the pulse and temperature, or the feelings of the patient. Wheeler[4] is in favour of early operative procedure in every case of appendicitis. If the rule to operate as soon as the diagnosis is made is always followed, some patients will be operated upon who would have recovered perfectly without operation. If medical treatment is always followed, a much larger number of patients will die who would have been saved by prompt operative interference. Moreover, the medically treated cases will include many which afterwards recur. If we reserve operative interference for cases whose symptoms imperatively demand it, we shall now and then lose a patient whose appendix has neglected to display danger signals before perforating or suppurating. Harte[5] reports a case of pyæmia due to appendicitis. All the symptoms pointed to the liver as the diseased organ, but post-mortem the appendix was found to be entirely destroyed, and its position occupied by a small pus cavity holding about three drachms of pus. The cæcum for several inches beyond its attachment to the appendix was gangrenous. There were some septic deposits in the lungs, and the liver contained a large number of metastatic abscesses.

**The mechanism of Perforation of the Appendix** has been the subject of experimental investigation by Roux[6], who concludes that the presence of foreign bodies is not alone sufficient to determine perforation, even if there be marked disturbance of the arterial circulation, and that these foreign bodies are tolerated as long as the mucous membrane is not inflamed. Evidently, however, a slight cold and consequent intestinal catarrh is all that is needed to make the inflamed mucous membrane attach itself, as it were, to the foreign body, which facilitates the production of gangrene. A further proof that perforation is not the result of the direct action of concretions is furnished by the distribution of the mortified patches in such a manner as to resemble a map, a fact which cannot be explained on the supposition of extreme mobility of the foreign body alone. It is, therefore, probable that the production of perforations is determined, not by the mechanical action of concretions, but rather by the vital action of microbes, the effect of the presence of the foreign body being merely to localise and hasten the process.

**Appendicitis Obliterans.**—Senn[7] draws attention to this hitherto undescribed form of appendicitis. He says: (1) Appendicitis obliterans is a comparatively frequent form of relapsing inflammation of the vermiform appendix; (2) it is characterised by progressive obliteration of the lumen of the appendix by the gradual disappearance of the epithelial lining and glandular tissue, and the production of granulation tissue from the sub-mucous connective tissue, which, by transformation into cicatricia tissue, starves out remnants of glandular tissue, and finally results in obliteration; (3) the obliterating process manifests a progressive tendency, and may finally result in complete destruction of all glandular tissue and obliteration of the entire lumen; (4) the incipient pathological changes occur either in the mucous membrane of the appendix, in the form of simple ulceration, or as an interstitial process following lymphatic infection; (5) the most constant symptoms which attend this form of appendicitis are relapsing acute exacerbations of short duration, moderate swelling at the seat of disease, and persistence of tenderness in the region of the appendix during the intermissions; (6) the process of obliteration may begin at the distal or proximal end, or at any place between, or it may commence simultaneously, or in succession at different points; (7) obliteration at the proximal side gives rise to retention of septic material, which finds an outlet through the lymphatics, and gives rise to non-suppurative lymphangitis and lymphadenitis; (8) circumscribed plastic peritonitis is an almost constant concomitant of appendicitis obliterans, and hastens the process of obliteration; (9) complete obliteration of the lumen of the appendix results in a spontaneous and permanent cure; and (10) in view of the prolonged suffering incident to a spontaneous cure by progressive obliteration, and the possible dangers attending it, a radical operation is indicated, and should be resorted to as soon as a positive diagnosis can be made.

[1] Med. Week, Sept. 7th, 1894, and Deut. Zeit. f. Chir. xxxviii. [2] Amer. Journ. of the Med. Sci., Oct., 1894, and Centr. f. Chir., 1893, No. 44. [3] Med. Record, New York, Oct. 6th, 1894, p. 425. [4] N.Y. Med. Journ., Nov. 3rd, 1894, p. 563. [5] Annals of Surgery, Oct., 1894, p. 422. [6] Med. Week, Oct. 19th, 1894. [7] Brit. Med. Journ., Oct. 6th, 1894, and Canad. Pract., July, 1894.

